# Effects of music therapy on cancer-related fatigue, anxiety, and depression in patients with digestive tumors: A protocol for systematic review and meta-analysis

**DOI:** 10.1097/MD.0000000000025681

**Published:** 2021-06-04

**Authors:** Xiaxia Chen, Qiuya Wei, Ruirui Jing, Yong Fan

**Affiliations:** Department of General Surgery, Lanzhou University Second Hospital, Lanzhou, Gansu, China.

**Keywords:** digestive tumor, music therapy, cancer-related fatigue, anxiety, depression, randomized controlled trial, meta-analysis

## Abstract

**Background:**

Digestive tumor is one of the most common cancers, its symptoms and treatment will bring patients with anxiety, depression and other negative emotions, and cause cancer-related fatigue. As a new complementary replacement therapy, music therapy can greatly reduce cancer-related fatigue, anxiety and depression, and achieve good clinical results, but there is a lack of evidence-based medicine. The purpose of this study is to evaluate the effect of music therapy on cancer-related fatigue, anxiety, and depression in patients with digestive tumors by meta-analysis.

**Method:**

Computer search of Chinese and English databases: Wanfang, VP Information Chinese Journal Service Platform, China National Knowledge Infrastructure, Chinese BioMedicine Literature Database and pubmed, embase, cochrane, web of science. A comprehensive collection of relevant studies on the effects of music therapy on digestive tract cancer-related fatigue, anxiety and depression, the retrieval time is from the date of establishment to March 2021. According to the inclusion and exclusion criteria, the literature is selected, the quality of the literature is evaluated and the data are extracted. The data are analyzed by meta-analysis.

**Result:**

The purpose of this study is to evaluate the effect of music therapy on digestive tract cancer-related fatigue, anxiety, and depression by European Organization for Research and Treatment of Cancer Quality of Life Core Questionnaire, Hamilton Depression Scale, and Hamilton Anxiety Scale.

**Conclusion:**

This study will provide reliable evidence-based evidence for the clinical application of music therapy in the treatment of digestive tract cancer-related fatigue and anxiety and depression.

**OSF Registration number:**

DOI 10.17605/OSF.IO/UR4GV.

## 1 Introduction

Digestive tumors are malignant tumors that occur in the epithelial tissue of the digestive system,^[1]^ including tumors of different digestive system organs: Esophagus, stomach, pancreas, liver, gallbladder, small intestine, large intestine, rectum, and anus. They are the most frequently diagnosed types of tumor in the world,^[2]^ accounting for 30% of tumor cases and 32% of tumor-related death.^[3]^ In 2018, the 2 most common digestive tumors, colorectal cancer, and gastric cancer, caused about 2.8 million new cases and 1.6 million deaths worldwide, respectively.^[4]^ Digestive tumor clinical manifestations are obstruction, mass, bleeding and pain, and other symptoms, some patients are even difficult to swallow, unable to eat, resulting in negative emotions such as anxiety and depression, and the quality of life continues to decline.^[5]^ The main treatment methods are operation and chemotherapy.^[6]^ Studies have shown that up to 90% of tumor patients have received chemotherapy.^[7]^ Adverse reactions after chemotherapy can lead to persistent and chronic oxidative stress, psychological stress, and psychological disorders, which will lead to cancer-related fatigue and reduce the quality of life of patients.^[8,9]^

As a new complementary and alternative therapy, music therapy is easily accepted by cancer patients.^[10]^ Music therapy treats patients’ physical or mental diseases through rhythm and tone, helps patients better express their emotions and promotes inner emotional communication.^[11]^ For cancer patients, music therapy is mainly used to promote relaxation, reduce anxiety, depression and other emotions, relieve pain, relieve physical discomfort, and related symptoms caused by treatment.^[12]^

At present, there are a number of randomized controlled trials (RCTs).^[13–16]^ The results show that music therapy can effectively improve the sleep quality of patients, relieve depression and anxiety, and improve the symptoms of fatigue. However, the sample size of many studies is small, and there are differences in intervention methods, intervention cycles, intervention time and other factors, and the literature quality and efficacy have not been systematically evaluated. Therefore, this study plans to systematically evaluate the effect of music therapy on cancer-related fatigue and anxiety and depression in patients with digestive tumor. So as to provide reliable evidence-based basis for the clinical application of music therapy for cancer-related fatigue and anxiety and depression in patients with digestive tumor.

## 2 Methods

### 2.1 Protocol register

This protocol of systematic review and meta-analysis has been drafted under the guidance of the preferred reporting items for systematic reviews and meta-analyses. And, this study has been registered on open science framework (OSF) (Registration number: DOI 10.17605/OSF.IO/UR4GV).

### 2.2 Ethics

Since all the data used in this study have been published, there is no need to collect personal information, so the approval of the Ethics Committee is not required. In addition, all data will be anonymously analyzed during the review process.

### 2.3 Eligibility criteria

#### 2.3.1 Types of studies

A RCT is included in the effects of music therapy on cancer-related fatigue, anxiety, and depression in digestive tumors. The literature language is limited to Chinese and English. There is no limit to the publishing area and time.

#### 2.3.2 Research objects

Patients are diagnosed as digestive tumor by pathology. Patients with hearing impairment are excluded. The nationality, race, gender, and age of the patients included in the study are not limited. There is no limit to the early, middle, and late stages of the tumor. The type of digestive tumor is not limited.

#### 2.3.3 Interventions

The intervention group is treated with music therapy combined with conventional antitumor therapy, while the control group is treated with conventional antitumor therapy.

#### 2.3.4 Outcome indicators

Main outcome: EORTC QLQ-C30, Hamilton Depression Scale, Hamilton Anxiety Scale.

Secondary outcome: Defecation condition, PSQI, Visual Analogue Scale.

### 2.4 Exclusion criteria

1. Repeatedly published literature.
2. The full-text literature cannot be obtained.
3. Literature in which the outcome index is not consistent.

### 2.5 Search strategy

Based on the principle of subject words combined with free words, a comprehensive search of Chinese and English databases such as PubMed, Embase, web of science, Cochrane Library, China National Knowledge Infrastructure, Wanfang, VP Information Chinese Journal Service Platform, and Chinese BioMedicine Literature Database is conducted from the establishment of the database to March 2021. Chinese search words mainly include: music therapy, digestive tumor, gastrointestinal cancer, etc; English search words include: music therapy, gastrointestinal neoplasms, gastrointestinal cancer, and so on. Take PubMed as an example, the retrieval strategy is shown in Table 1.

**Table 1:** PubMed database retrieval strategy.

Number	Search terms
#1	Music therapy[MeSH]
#2	Music therapy [Title/Abstract]
#3	#1OR#2
#4	gastrointestinal neoplasms [MeSH]
#5	gastrointestinal neoplasms [Title/Abstract]
#6	gastrointestinal cancer[Title/Abstract]
#7	gastrointestinal tumor [Title/Abstract]
#8	#4OR#5OR#6OR#7
#9	#3AND#8

### 2.6 Data screening and extraction

**Figure 1:**
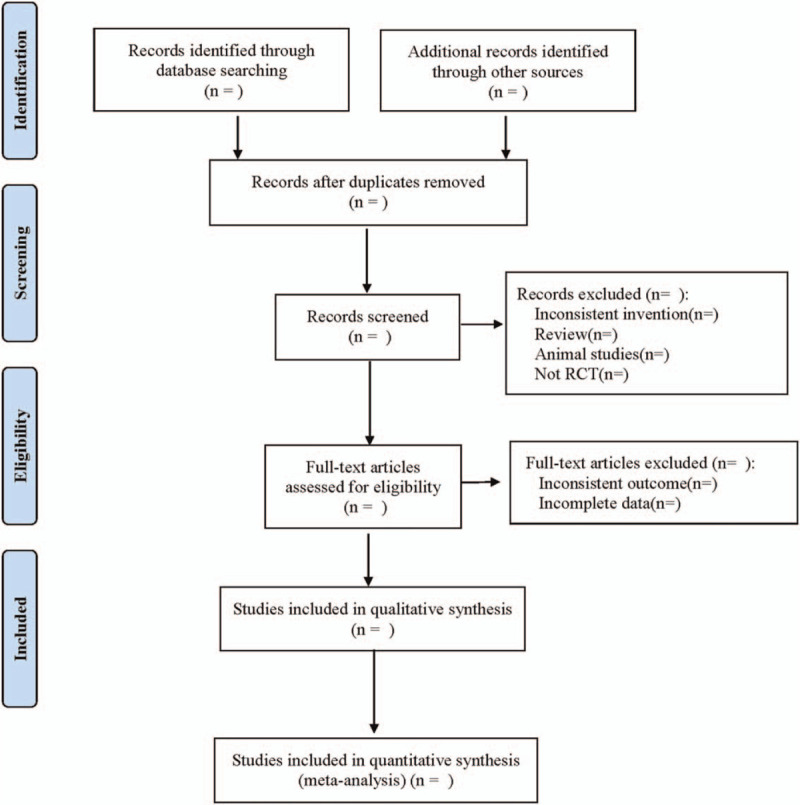
Flow diagram.

Before the formal screening, 2 articles from the retrieval results are randomly selected for pretest to unify the understanding of the screening staff and further improve the inclusion and exclusion criteria. The 2 authors independently screen the literature according to the title and abstract, and download the full text of the screening results for the second screening. If there are any differences in the screening process, an agreement can be reached by discussing or referring to the opinions of a third party. According to the unified data collection table, the data are extracted by 2 researchers independently. The items of the data collection table mainly include: the basic situation of the experiment, such as the author and year of the literature, the grouping and intervention of the research objects, the measurement indicators of the results, the methodological quality of the study, and so on. The screening process is shown in Figure 1.

### 2.7 Literature quality evaluation

The research quality evaluation is carried out according to the Cochrane Manual for Systematic Evaluation of Interventions. The contents of the evaluation include:

1. whether the correct random method is reported;
2. whether the allocation is hidden;
3. whether the blind method is used;
4. whether there is an explanation for the object of withdrawal and loss of follow-up;
5. whether there are selective reporting results;
6. whether there are biases from other sources.

The evaluation results are divided into: low risk, high risk, unclear. Two persons check the evaluation results, and if they have different opinions, they will discuss with the third evaluator and reach a consensus.

### 2.8 Statistical analysis

#### 2.8.1 Data analysis and processing

Meta-analysis of the data is performed using RevMan 5.3 software. First of all, Chi-Squared test is used to determine whether there is heterogeneity between studies. If *I*^*2*^ *≤* 50% *P* *≥* .1, multiple similar studies can be considered to be homogeneous. Fixed effect model is selected for meta-analysis. If *I*^*2*^ *>* 50% *P* *<* .1, but there is only statistical heterogeneity, and when it is clinically judged that there is consistency among the studies and needs to be merged, the random effect model can be selected to make a descriptive analysis of the data that cannot be combined. Continuous data are expressed by mean difference, the binary data are expressed by risk ratio, and the 9.5% confidence interval is reported.

#### 2.8.2 Dealing with missing data

If there is missing data in the article, contact the author by phone or email and improve the data before analysis. If the author cannot be contacted, or if the author has lost the relevant data, the meta-analysis will not be performed.

#### 2.8.3 Subgroup analysis

Subgroup analysis is carried out according to the age of young, middle-aged, and elderly. Subgroup analysis is carried out according to gender male and female. Subgroup analysis is carried out according to different types of digestive tumors; subgroup analysis is carried out according to the early, middle, and late stages of the disease.

#### 2.8.4 Sensitivity analysis

The included studies are deleted one by one to evaluate the impact of a single study on the overall results.

#### 2.8.5 Assessment of reporting biases

The funnel plot is used to understand whether there is publication bias in the article.

#### 2.8.6 Evidence quality evaluation

According to the Grading of Recommendations Assessment, Development, and Evaluation rating table, the evidence quality and the reliability of the results are evaluated from 5 aspects: bias risk, consistency, directness, accuracy, and publication bias, which are divided into 4 grades: high quality, medium quality, low quality, and very low quality.

## 3 Discussion

In recent years, digestive tumor has attracted wide attention all over the world as a kind of physical and mental disease. In general hospitals in China, anxiety and depression, and somatic symptoms are caused by cancer often merge and influence each other.^[17]^ Cancer-related fatigue is a persistent, subjective fatigue, or feeling of exhaustion associated with tumor or tumor therapy, and has nothing to do with recent activities.^[18]^ The incidence of fatigue in patients with digestive tumor is high, and the degree is severe.^[19]^ A study^[20]^ found that the incidence of cancer-related fatigue during and after treatment was 25% to 99% and 29% to 38%, respectively. Sleep, appetite, and pain have the greatest influence on cancer-related fatigue.^[21]^

Music-guided imagination can reduce blood pressure and plasma cortisol levels, reduce anxiety and depression, and improve the quality of life and well-being of cancer patients.^[22]^ In the process of tumor, psychological stress, and sleep disturbance can promote an inflammatory response, imbalance of hypothalamus-pituitary-adrenal axis and decrease of immune surveillance function.^[23]^ Music can regulate the stress response of hypothalamus-pituitary-adrenal axis^[24]^ and has a therapeutic effect on mental diseases such as depression.^[25]^ At present, music therapy plays a role in clinical applications such as neuropsychiatric system, cardiovascular system, digestive system, cancer, and surgery.^[26]^ However, there is no standard program that can be implemented in music therapy worldwide.^[27]^ The American Music Association proposes that all types of music may play a role in changing patients’ lives, and music should be chosen according to the preferences and needs of patients.^[28]^ The implementation time is generally 15 minutes to 1 hour, which can be either a single course of treatment or multiple courses of treatment.^[29]^

We hope to conduct a meta-analysis of RCTs of cancer-related fatigue and anxiety and depression in digestive tumors through the existing music therapy, so as to provide reliable evidence-based basis for the clinical application of music therapy in digestive tumors. However, due to the small sample size, music therapy is difficult to set up blindness. At the same time, there are differences in the personnel, duration, and methods of music therapy in various studies. Large sample and high-quality research can be carried out on the basis of standardized music therapy to provide evidence for the standardized implementation of music therapy. Due to the limitation of language ability, we only search English and Chinese literature and may ignore studies or reports in other languages.

## Author contributions

**Data curation:** Xiaxia Chen, Qiuya Wei.

**Funding acquisition:** Yong Fan.

**Investigation:** Ruirui Jing.

**Software:** Ruirui Jing.

**Writing – original draft:** Xiaxia Chen, Qiuya Wei.
